# Psychomotor Development in Cri du Chat Syndrome: Comparison in Two Italian Cohorts with Different Rehabilitation Methods

**DOI:** 10.1155/2016/3125283

**Published:** 2016-11-28

**Authors:** Andrea Guala, Marianna Spunton, Fabio Tognon, Marilena Pedrinazzi, Luisa Medolago, Paola Cerutti Mainardi, Silvia Spairani, Michela Malacarne, Enrico Finale, Mario Comelli, Cesare Danesino

**Affiliations:** ^1^Department of Pediatrics, Castelli Hospital, Verbania, Italy; ^2^Associazione Bambini Cri du Chat (ABC), S. Casciano in Val di Pesa, Florence, Italy; ^3^Child Neuropsychiatry Unit, Department of Brain and Behavioural Sciences, University of Pavia, Pavia, Italy; ^4^Laboratory of Human Genetics, E.O. Hospital Galliera, Genova, Italy; ^5^Department of Brain and Behavioural Sciences, University of Pavia, Pavia, Italy; ^6^Department of Molecular Medicine, University of Pavia and IRCCS, S. Matteo, Pavia, Italy

## Abstract

The Cri du Chat syndrome (CdC) is a rare genetic disorder caused by variable size deletions of the short arm of chromosome 5 (5p−). It is well known that home-reared patients show better performances as compared to institutionalised cases, and it was reported that continuous educational intervention can ameliorate their performances. To assess the efficacy of educational intervention and to develop new CdC oriented programs of rehabilitation, we compare the results obtained for many developmental skills in two groups of CdC patients undergoing two different rehabilitation programs. Using data on the development of a group of CdC patients obtained by validated Italian translation for the Denver Developmental Screening Test II, we compared a group of 13 patients undergoing an educational program developed for CdC patients, the Mayer Project (MP), with a second group of 15 cases in whom caring was not specifically oriented. A positive impact of the MP was reported by parents, observing an improvement in social skills obtained, even if no significant differences were observed when the items of the Denver Test are studied. The need for personalized care in CdC patients and the choice of different methods to compare the results are also discussed.

## 1. Introduction

The Cri du Chat syndrome (CdC) is a rare genetic disorder caused by variable size deletions of the short arm of chromosome 5 (5p−). The incidence ranges from 1:15,000 to 1:50,000 live-born cases. Main clinical features include high-pitched cry, microcephaly, broad nasal bridge, epicanthal folds, micrognathia, and severe psychomotor retardation. Cardiac and renal malformations may be also found. A molecular cytogenetic map of the deletion has been tentatively established [1].

As the degree of mental retardation is significant, it causes relevant problems in caring for the patients. It is likely that many of the developmental and behavioural problems of CdC can be linked to the size of the deletion and to patient-specific genome variations.

It is now well known that home-reared patients show better performances as compared to institutionalised cases, and it was reported that continuous educational intervention can ameliorate their performances [2, 3].

To assess the efficacy of educational intervention and to develop new CdC oriented programs of rehabilitation, it is important to describe their developmental steps. In fact, developmental charts have been developed (gross motor, fine motor, speech, and personal/social) collecting data from 84 Italian patients [4].

We compare the results obtained for many developmental skills in two groups of CdC patients in whom rehabilitation was performed according to two different methods.

## 2. Materials and Methods 

To evaluate gross motor, fine motor, speech, and personal/social parameters, we used the same methods used for collection of developmental data [4], and we studied two groups of patients, all born in 2005 or later, to avoid large time-related differences in care. Both groups were scattered all over Italy.

The ABC (Associazione Italiana Bambini Cri du Chat) recruited over several years 28 patients whose ages were between 6 and 12 months and offered their families the possibility of being followed up by the same expert in special education (ED) who suggested the type of educational intervention.

The ED offered to contact the families twice a year, for a 2–4-day home visit first, followed after 6 months by evaluation of the compliance to the given suggestions. During the first visit, the ED evaluated the management of patients (by their families and different local operators, as nurses, physiotherapists, speech therapists, teacher, professional educator, etc.) as related to motor development and training for everyday social life. ED then provided patient-specific suggestions to improve the ongoing family and local professional care. This educational program is from now on referred to as Mayer Project (MP).

For each patient, the developmental profile was assessed and a customized daily program including sensory stimuli and motor opportunity was developed.

Children unable to walk were kept on the floor in a prone position if not involved in any other activity (eating, sensory stimuli, or snuggling).

Sensory stimuli, for all children, included touch, hearing, view, and balance training.

Touch included wiping, superficial touching, rubbing, kneading, and vibration stimuli. All stimuli were extended over the whole body excluding the face and genitalia and were repeated five times a day, each lasting 1 minute, for several months, until reaching sufficient correct perception of the stimulus.

Touching stimuli were intended to improve body perception and motor performances and to reduce self-injury (biting, scraping, and prickling) [5].

Hearing stimuli were offered to improve hearing mediated attention and word comprehension. At least four times a day exercises for sound localisation and general and onomatopoeic sounds reproduction were performed, selected according to patient's age.

Visual stimuli were offered four times a day showing and at the same time naming flash cards with unambiguous high resolution images. The flash cards were changed weekly. These stimuli are intended to develop at the same time hearing and visual attention. As in older CdC patients, balance difficulties and ataxic gait [2, 6] were observed; exercises were proposed to improve both passive and active balance and to prevent balance difficulties appearing with age. In younger patients, swinging on a blanket carpet, seesaw, and swivel chair was proposed; in older patients, exercises include rolling on a carpet, flips, and walking on a rough path. These stimuli are also intended to improve visual and motor coordination. Crawling in the prone position in cross pattern was the initial motor exercise suggested for all patients: 70 to 400 meters/day was requested to be done according to each patient level of motor abilities. Crawling was intended to improve whole body musculature, eye control, swallowing, breathing, and motor coordination. The relevance of crawling and the possible problems arising when it is not provided were discussed by Zachry and Kitzmann [7]. The costs of MP were supported by the ABC, and 13 families accepted following this program; the children started to be followed up at ages between 6 and 12 months. The remaining 15 patients, “non-Mayer Project” (non-MP), underwent rehabilitation programs as proposed locally by different operators and included for all patients physiotherapy, logopedic therapy, and neuropsychomotor therapy of developmental age. All families were also requested to fill forms in which specific items (*n* = 88) related to gross motor, fine motor, speech, and personal/social motor development are described using the validated Italian translation for the Denver Developmental Screening Test II [8]. We used this version of the Denver Test in order to better compare new data with those previously collected [4]. The form to be filled by the families is available upon request; details for each item in each patient are included as additional material. The number of patients, for whom the different items in the forms can be evaluated, varies as a function of their age: as the study started in 2005, at the time of data collection, some cases were 9 years old while others were 2 years old (spring 2012). The ages at which each patient achieved each item, or some subgroups of them (motor activities, visual activities, and eye-hand coordination; preverbal, verbal, and social interaction; self-care; and autonomy), were compared between MP and non-MP, using Kaplan-Meyer curves (Figure 2). In all patients, the clinical diagnosis of CdCS was confirmed by standard karyotype (G banding, 550 bands' resolution).

## 3. Results

Demographic and cytogenetic features of the two groups of CdCS patients are entered in Table 1. The two groups failed to show significant differences for age (*p* < 0.33), sex (*p* < 0.27), gestational age at birth (*p* < 0.9), and chromosomal break point distribution (*p* < 0.46).

**Figure 1:**
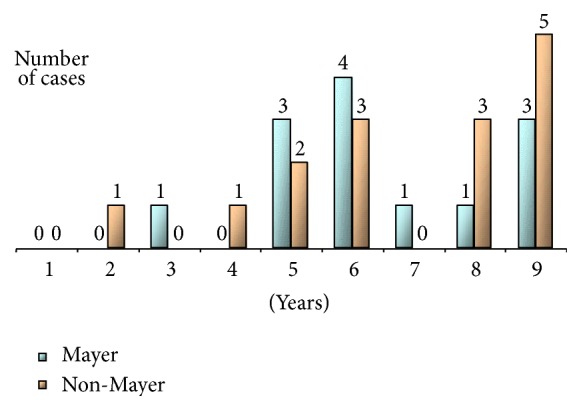
Age distribution of patients included in Mayer and non-Mayer Project.

**Figure 2:**
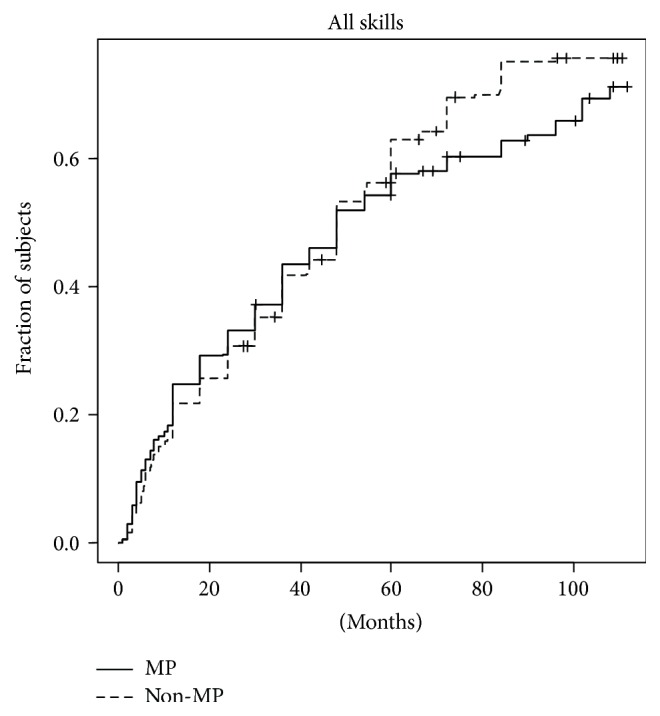
Kaplan-Meyer curves for age of achievements of all items included in the validated Italian translation for the Denver Developmental Screening Test I. MP: Mayer Project; non-MP: non-Mayer Project.

The comparison of ages at which each of the 88 items [4] was reached in the two groups (MP and non-MP) failed to show any significant difference (Figure 1).

We then grouped the 88 items in six subgroups (motor performances, eye-hand coordination, preverbal performances, verbal performances, social interaction, and self-care) and again no significant differences were observed (data not shown).

## 4. Conclusions

Data as to dysmorphic features and neurological profile in CdC are largely available [1, 4], while data concerning methods and efficacy of rehabilitation are lacking or related to single case report [9]. In fact, Sigafoos et al. [10] suggested how to develop rehabilitation programs, which should include strategies whose efficacy in children or adults with different types of neuromotor delay has already been demonstrated.

To the best of our knowledge, no CdC tailored rehabilitation programs are to date available even if already in 1980 Wilkins et al. [3] stated that “achievement levels were influenced favourably by the early introduction of special education.” The “Cri du Chat supporting group” from UK provides “Handbook for Parents and Professionals” containing a large amount of practical advice for caring and improving the development of skills in CdC patients [11], but there is no single patient tailored program of education. A similar handbook is provided by the ABC [12].

In 2008, Pizzamiglio et al. [13] reported a single case in whom improvement of visual-motor coordination was obtained by a computer based program in which the patient was requested to “touch a picture on the screen with a coordinated hand movement to obtain the appearance of a new picture.”

The method described in this report shares some similarities with the visual stimulation offered in the MP program. A further CdC patient, with mild developmental delay, when investigated for emotional competence, demonstrated a performance similar to age matched controls [14], demonstrating that personalized training may be beneficial. So, the availability of new computer based methods if joined to personalized educational training is likely to significantly improve the final outcome of performances in CdC patients.

A first step to pursue this goal could be an accurate and detailed description of their psychomotor development, and this was done as reported by Cerruti Mainardi et al. [4].

The personal experience of a group expert in special education (FaTo, MaPe, and LuMe) led to the proposal to ABC of the method described above (http://www.criduchat.it/documents/ABC-Criduchat-Technical-aspects-Educational-Guidelines-EN-web.pdf). At present, the only possibility of evaluating the efficacy of the proposed method was to compare available data about psychomotor development between the two groups of cases, MP and non-MP. As reported in the methods, inclusion into the project was on a parental voluntarily basis only, and the patients were not randomised. The comparison failed to demonstrate significant differences between the two groups.

However, parents consistently reported improvement in everyday behaviour, as “general self-care,” “to be able to wait,” “to be with people,” or “to sit quietly for lunch.” Some older patients also experienced skiing or horse riding. In addition, parents involved in the MP program have been instructed to speak with their children asking very clear-cut questions, and they are almost sure to be well understood and to obtain adequate responses; to achieve this result, they usually subdivide complex duties into simpler subitems that the child is then able to understand and perform. Cooperation with teachers in MP group also resulted in a clear increase of their awareness about the learning skills present in CdC patients.

The educational needs of children with CdC, according to their parents' opinions, have been discussed by Pituch et al. [15], who reported parents with high priority personal safety skills.

Child frequency and severity of behavioural problem were reported by Cornish and Bramble [16] as the best determinant for familial stress.

To evaluate any improvement on these behavioural skills, the test reporting on psychomotor development [4], which of course was intended to describe the neurological impairment, is not the best choice, and this is likely the explanation for the lack of significance.

The educational program “MP” is still ongoing, and collection of data based on different scales, to obtain a quantitative evaluation of parental observations about improvements obtained by the MP, is in progress.

The extensive clinical heterogeneity reported by Wilkins et al. [17] and the observation by Albano et al. [14] that in some mildly affected cases emotional competences may be comparable to controls stress the relevance of developing new and personalized methods of education in CdC patients.

## Figures and Tables

**Table 1:** Demographic and cytogenetic features of the two groups of CdCS patients.

	Mayer Project		Non-Mayer Project	
Number of patients	13		15	
M/F	9M	4F	7M	8F
Mean age at data collection, years (range)	6.5 (3–9)		7 (2–9)	
Gestational age at birth, weeks (range)	36.83 (32–40)		37.13 (28–40)	
Associated malformations	—		—	
Head circumference at term or corrected as to 40-week gestational age	34		31.5	
Standard karyotype: 5 p−				
Break point in band				
15.2	1		2	
15.1	2		—	
14	8		7	
13	1		1	
Interstitial deletion	1		3	
Unbalanced translocation	—		2	

## References

[1] Cerruti Mainardi P. (2006). Cri du Chat syndrome. *Orphanet Journal of Rare Diseases*.

[2] Cornish K. M., Pigram J. (1996). Developmental and behavioural characteristics of cri du chat syndrome. *Archives of Disease in Childhood*.

[3] Wilkins L. E., Brown J. A., Wolf B. (1980). Psychomotor development in 65 home-reared children with cri-du-chat syndrome. *The Journal of Pediatrics*.

[4] Cerruti Mainardi P., Guala A., Pastore G., Pozzo G., Dagna Bricarelli F., Pierluigi M. (2000). Psychomotor development in Cri du Chat syndrome. *Clinical Genetics*.

[5] Connors E. R. (2008). *Self Injury: Psychotherapy with People Who Engage in Self-Inflicted Violence*.

[6] Colover J., Lucas M., Comley J. A., Roe A. M. (1972). Neurological abnormalities in the ‘cri-du-chat’ syndrome. *Journal of Neurology, Neurosurgery, and Psychiatry*.

[7] Zachry A. H., Kitzmann K. M. (2011). Caregiver awareness of prone play pecommendations. *American Journal of Occupational Therapy*.

[8] Frankenburg W. K., Dodds J., Archer P., Shapiro H., Bresnick B. (1992). The Denver II: a major revision and restandardization of the Denver Developmental Screening test. *Pediatrics*.

[9] Erlenkamp S., Kristoffersen K. E. (2010). Sign communication in Cri du chat syndrome. *Journal of Communication Disorders*.

[10] Sigafoos J., O'Reilly M. F., Lancioni G. E. (2009). Cri-du-chat. *Developmental Neurorehabilitation*.

[11] Cornish K. M., Oliver C., Standen P., Bramble D., Collins M. (2003). *Cri du Chat Syndrome: Handbook for Parents and Professionals*.

[12] Nardi S. http://www.criduchat.it/documents/ABC-Criduchat-Technical-aspects-Educational-Guidelines-EN-web.pdf.

[13] Pizzamiglio M. R., Nasti M., Piccardi L., Vetturini C., Morelli D., Guariglia C. (2008). Visual-motor coordination computerized training improves the visuo-spatial performance in a child affected by Cri-du-Chat syndrome. *International Journal of Rehabilitation Research*.

[14] Albano S., Piccardi L., Pizzamiglio M. R., Volpe C., Damico S. (2013). Narrative discourse and sociocognitive abilities of a child with cri-du-chat syndrome. *Journal of Genetic Psychology*.

[15] Pituch K. A., Green V. A., Didden R. (2010). Educational priorities for children with Cri-Du-Chat syndrome. *Journal of Developmental and Physical Disabilities*.

[16] Cornish K., Bramble D. (2002). Cri du chat syndrome: genotype-phenotype correlations and recommendations for clinical management. *Developmental Medicine and Child Neurology*.

[17] Wilkins L. E., Brown J. A., Nance W. E., Wolf B. (1983). Clinical heterogeneity in 80 home-reared childrenwith cri du chat syndrome. *The Journal of Pediatrics*.

